# The Proper Weight

**Published:** 1890-08

**Authors:** 


### THE PROPER WEIGHT.

Growth is very irregular in children and young people generally; perhaps two inches may be gained in two months, and for the next ten months not another inch, even up to the age of ten or twelve years.

While growth is thus rapid fatigue is readily reduced; during the pause weight is gained and work or training can go on again.

As a general rule a child in the fourth year should be 3 feet high, and weigh more than 28 pounds; in the sixth year, 3½ feet high, and weigh 42 pounds; in the eighth year, 4 feet high and 56 pounds in weight; at twelve years old, 5 feet in height and 70 pounds in weight is a fair average.

At the term of adolescence 28 pounds should be added for a gain of 3 or 4 inches in height, 112 pounds is about the average weight for 5 feet 6 inches; 126 pounds for 5 feet 8; 140 pounds for 5 feet 10; 154 pounds for 5 feet 11, and 168 pounds for 6 feet.

